# Investigation of Pharmacological Mechanisms of Yinhua Pinggan Granule on the Treatment of Pneumonia through Network Pharmacology and *In Vitro*

**DOI:** 10.1155/2022/1602447

**Published:** 2022-11-02

**Authors:** Liang Jin, Yumei Zhang, Jiehong Yang, Huifen Zhou, Gaozhi Jia, Yu He, Haitong Wan

**Affiliations:** ^1^School of Life Science, Zhejiang Chinese Medical University, Hangzhou, China; ^2^Department of Oncology, Shanghai East Hospital, School of Medicine, Tongji University, China; ^3^College of Basic Medical Science, Zhejiang Chinese Medical University, Hangzhou, China; ^4^National & Local Joint Engineering Research Center of Orthopedic Biomaterials, Peking University Shenzhen Hospital, Shenzhen, China; ^5^College of Pharmaceutical Science, Zhejiang Chinese Medical University, Hangzhou, China

## Abstract

Yinhua pinggan granule (YHPGKL), a traditional Chinese medical compound, could treat pneumonia. Although previous studies demonstrated the protective and therapeutic effects of YHPGKL on pneumonia, its potential molecular mechanisms and its effective components are still elusive. Herein, we performed a network pharmacology analysis to determine the possible signaling pathways involved in the protective effects of components of YHPGKL. A total of 119 components and 257 target proteins of YHPGKL were identified, among which 117 effective components interacted with 113 proteins related to pneumonia. Then, a compound-effective component-target protein network was established to screen the effective hub components. The top three effective components, namely luteolin, kaempferol, and quercetin, were selected. Moreover, Gene Ontology (GO) and Kyoto Encyclopedia of Genes and Genomes (KEGG) enrichment analysis of 113 proteins revealed a significant enrichment term associated with host immune and anti-infectious responses. Furthermore, by constructing a protein-protein interaction network between common proteins, ten hub proteins were identified, among which three hub components hit eight proteins. A further molecular docking analysis confirmed that the three effective hub components had a good affinity with six hub proteins. Eventually, the interactions were further visualized and screened on account of an infectious macrophage model *in vitro*. The results noted that three components could inhibit proinflammatory related hub genes but had no effect on survival-related hub genes. Thus, the three effective hub components and corresponding hub genes may play essential roles in the treatment of YHPGKL on pneumonia.

## 1. Introduction

According to the Global Burden of Disease report, respiratory infections are second threatened to human death. Pneumonia, the most common respiratory disorder, causes extremely high morbidity and mortality, particularly in young children (<5 years old) and older people (>65 years old) [1, 2]. Pathologically, pneumonia exhibits symptom of inflammation in the alveoli, terminal airway, and lung interstitium blamed on invading various bacterium, viruses, or other pathogens [3]. In the clinic, the patients with pneumonia suffer from acute onset fever, chills, or/and cough; other complications, including fatigue, anorexia, and pleuritic chest pain, are also reported [4]. The high incidence and mortality of pneumonia led to an overburden of the health system for most countries as well as a severe threaten to human life [5].

So far, antibacterial, antiviral, antipyretic, antihistamine, and supportive therapies are beneficial to pneumonia treatments [4]. However, multidrug-resistant (MDR) bacteria and viruses frequently spread over hospitals, limiting the efficiency of pneumonia treatment. For instance, methicillin-resistant S. aureus (MRSA) is more dangerous than methicillin-sensitive S. aureus due to higher mortality [6]. Additionally, the side effects of drugs (e.g., tetracycline resulting in yellow of teeth, erythromycin leading to increased intestinal peristalsis, and orange discoloration of the skin after treatment with rifampin) inevitably hamper the treatments for pneumonia patients [7–9]. Thus, it is essential to find new approaches with better therapeutic effects and fewer side effects than current medicines.

Traditional Chinese Medicine (TCM) has been used for thousands of years thanks to its good efficacy, few side effects, and special characteristics [10]. TCM compounds are composite of several natural herbs on account of TCM theory and the scientific mechanisms of which unique therapeutic efficiencies of TCM interact with various effective components and target molecules [11]. Ma Huang Tang (Ephedra Decoction), a compound out of the Treatise of Febrizle Disease written by Zhang ZhongJing, a famous Chinese traditional doctor living 2000 years ago, has been used to attenuate symptoms of cold, fever, cough, and asthma [12, 13]. The modern clinical trials showed that modified Ma Huang Tang could protect against respiratory infection [14]. Based on the Ma Huang Tang compound, our group invented the Yinhua pinggan granule (YHPGKL) comprised of Lonicerae Japonicae Flos (JYH), Licorice/Glycyrrhizae (GC), Puerariae Lobatae Radix (GG), Ephedra Herba (MH), Polygoni Cuspidati Rhizoma Et Radix (HZ), and Amygdalus Communis Vas (XR) [15]. As an improved TCM, these components of YHPGKL have been reported to function as anti-infection, antivirus, antioxidant, and antiapoptosis. For example, both JYH and MH showed anti-infection and antivirals among influenza virus infection via inhibiting virus replication [16, 17]. Zhou et al. demonstrated that GC prevented renal tubular cells from oxidative damage by regulating antioxidant signaling [18]. Furthermore, Chang et al. found that GG enhanced the immune activity of macrophages by using immunosuppressive mice [19]. Additionally, literate reported that GC could inhibit the expression of proinflammatory cytokines induced by various pathogens [20]. In our previous reports, YHPGKL attenuated the symptoms of mice with pneumonia by regulating Toll-like receptors (TLR) signaling pathway, e.g., TLR-4-myeloid differentiation factor (MYD) 88-TNF receptor associated factor (TRAF)/nuclear factor kappa-B (NF-*κ*B), *in vivo* and *in vitro* [15, 21, 22]. Most recently, our group found YHPGKL prevented apoptosis of lung tissue from mice with pneumonia through suppressing apoptotic related proteins such as BAX and Caspase-3 [23]. Although previous data showed YHPGKL had a good therapeutic effect on pneumonia, giving the compound having 6 herbs and hundreds of active components, the potential interaction of effective components and molecular targets are still not clear, resulting in elusive pharmacological mechanisms.

The network pharmacology provides a practical approach that can transfer one target, one drug analysis of TCM to the multitargets, multicomponents analysis, accelerating the scientific application of TCM [24]. Since the TCM usually comprises various herbs containing hundreds of thousands of components, it is difficult for researchers to screen out critical components *in vitro* and *in vivo* due to the high cost and work overload. Therefore, this study investigates the relationships between effective components of YHPGKL and target proteins related to pneumonia through network pharmacology analysis. By means of database collection, Gene Ontology (GO) and Kyoto Encyclopedia of Genes and Genomes (KEGG) enrichment analysis, protein-protein interaction network (PPI) establishment, molecular docking experiment, and *in vitro* study, we extracted some hub components from YHPGKL. We found some critical proteins related to pneumonia, which may be potential targets for the treatment of YHPGKL.

## 2. Materials & Methods

### 2.1. Active Components of YHPGKL

Since both oral bioavailability (OB) and drug-likeness (DL) are the critical factors in regulating drugs absorption in the body, we screened out all components of YHPGKL using the Traditional Chinese Medicine System Pharmacology (TCMSP, Version 2.3) by imputing the keywords “jinyinhua,” “gegen,” “huzhang,” “mahuang,” “xingren,” and “gancao,” separately [24]. The TCMSP is a unique systems pharmacology platform of herbal medicines that analyzes the relationships between herbs, targets, and diseases, which has been widely used in TCM. The database includes hundreds of thousands of information on chemicals, targets, and components-target networks. Besides, the pharmacokinetics of natural components, including OB, DL, intestinal epithelial permeability, blood-brain-barrier, and aqueous solubility, were stored in the TCMSP. Then, we selected active components of YHPGKL with the criteria OB>30% and DL>0.18 [25]. The structure information of active components was acquired from the PubChem database [26]. Furthermore, the active components of YHPGKL-related proteins were obtained in the drug bank database; they were further standardized by the UniProt database, which provides comprehensive, high-quality protein functional information [26, 27].

### 2.2. Targets of Pneumonia Analysis

Since the potential therapeutic symptoms of YHPGKL focus on various pathological factors induced inflammation of lung, we thought pneumonia was able to cover up the most of above-mentioned symptoms and related pathomechanisms to help us find out more universal therapeutic targets. Proteins related to pneumonia were collected from the Gene Cards database [28] and OMIM database [29], using “pneumonia” as the keyword. Both Gene Cards and OMIM are searchable, integrative databases offering comprehensive, accurate information on relationships between diseases and human genes, which are widely used in medical bioinformatics. After removing the duplicate results, we collected 1423 pneumonia-related targets from both databases. Finally, these targets were further validated by Comparative Toxicogenomics database (CTD). These validated targets were standardized using the UniPort database.

### 2.3. Protein-Protein Interactions (PPI) Network Analysis

After screening out cotarget proteins between active components and pneumonia, we analyzed their relationships using the STRING database. The STRING is a popular database of known and predicted protein-protein interactions. The interaction contains direct (physical) and indirect (functional) associations; they come from computational prediction, knowledge transfer amid organisms, and interactions aggregated from other databases. In this study, the STRING database analyzed the potential targeted proteins, with settings of a confidence score ≥ of 0.4 and the Organism of “*Homo sapiens*” [30].

### 2.4. Cluster Analysis

Based on PPI analysis, we made an MCODE analysis, clustering a given network based on the topology to find densely connected regions, to extract similar nodes and protein complexes to perform clusters using the Metascape database with settings of physical core (1152473,19%), Min Network Size:3, Max Network Size:500 [31]. The Metascape is a well-known database providing a broad spectrum of bioinformatics tools to analyze multisystems-level datasets [31].

### 2.5. Visualization of Network and Identification of Hub Protein and Hub Components

We kicked out active components that did not target pneumonia-related proteins according to previous prediction and screening results. Then, the data were imputed into the Cytoscape 3.7.1 software (Cytoscape) for network construction [32]. The Cytoscape software, as open-source software, functions to visualize, analyze, and model to establish the network, which is usefully performed in the bioinformatics analysis. Herein, we set up a “herbs-effective components-targets” network, among which hub components were selected based on the degree value.

To screen out hub protein, based on the PPI analysis, we imputed PPI results into Cytoscape and rebuilt a new PPI network which visualized in line with degrees (the size and brightness of protein) and edges (the size and brightness of rope). Then, we picked up hub proteins according to the degrees of betweenness centrality and closeness centrality.

### 2.6. Gene Ontology (GO) Enrichment and Kyoto Encyclopedia of Genes and Genomes (KEGG) Pathway Analysis

GO annotation is used to define the functions of genes based on biological process (BP), cell components (CC), and molecular functions (MF). KEGG is a database that integrates genome, chemistry, and system function information. Therefore, the acquired target proteins for pneumonia were imputed into Metascape to get GO and KEGG functional enrichment analysis (*P* < 0.05).

### 2.7. Effective Hub Components and Hub Target Proteins Interaction Analysis

The chemical structures of effective hub components were obtained from the TCMPS database. The crystal structures of hub target proteins were acquired from the RCSB BDB database that provides access to 3D structure data for large biological molecules via Internet information portal downloadable files [33]. Then, the AutoDock 4.2.6 software was used to remove ligand, water molecular, added polar hydrogen atoms, and charges to the protein crystal structures, respectively [34]. The interaction of components and proteins was evaluated using the Discovery Studio software [35].

### 2.8. Cell Culture and Treatment

THP-1 cells, after stimulation into THP-1-derived macrophages (referred to as macrophages), were selected for assay due to their similarity to human primary macrophages [36]. Cells were acquired from the Shanghai Institutes for the Biological Sciences, Chinese Academy of Sciences, and cultured in RPMI 1640 medium supplemented with 10% fetal bovine serum (FBS) and 1% Penicillin & streptomycin (PS). Macrophages were seeded in a dish at a 1×10^6^/ mL concentration with various concentrations of luteolin, kaempferol, or quercetin (Aladdin, China) in the presence or absence of 1 *μ*g/mL Phosphate polysaccharide (LPS, Biosharp, China) for 24 h, respectively. Then, the THP-1 cells were challenged with 100 ng/mL phorbol 12-myristate 13-acetate (PMA, Sigma, USA) for 48 h to differentiate into macrophages.

### 2.9. Cytotoxicity Assay

To assess the cytotoxicity of luteolin, kaempferol, or quercetin, macrophages were seeded into 96-well plates along with luteolin, kaempferol, or quercetin at different concentrations for 24 h, respectively. Then, 10 *μ*l CCK-8 solution (Biosharp, China) was supplemented and incubated for another 2 h, followed by a microplate reader measurement (BioTek, Winooski, VT, USA) at a wavelength of 450 nm.

### 2.10. Real-Time Quantitative PCR (RT-qPCR)

Total RNA was isolated using the TRIzol reagent (Invitrogen, USA), and cDNA was synthesized with ReverTra Ace qPCR RT kit (Toyobo, Japan) according to the manufacturer's instructions, respectively. The RT-qPCR assay was performed with SYBR Green Real-Time PCR Master Mix (Toyobo, Japan). The target primer sequences are as follows:

IL6: 5′-CCACTCACCTCTTCAGAAC-3′, 3′-CTTTGCTGCTTTCACACAT-5′.

TNF: 5′-GTGATCGGCCCCCAGAGGGA-3′, 3′-CACGCCATTGGCCAGGAGGG-5′. AKT1: 5′-CTCCTGAGGAGCGGGAGGAGTGG-3′,3′-GTCCACTCCTCCCGCTCCTC A GGA-5′.

MMP9: 5′-TTCTCCAGAAGCAACTGTCC-3′, 3′-TAGGTG ATGTTGTGG T G GTG-5′. GAPDH: 5′-GGAGAAGGCTGGGGCTCAT-3′, 3′ TGATGGCATGGACT G TG GTC-5′. CAPASE3:5′-GCAGCAAACCTCAGGGAAAC-3′, 3′-TGTCGGCATA CTGT T TCAG C A-5′.

### 2.11. Statistical Analysis

Data are visualized as the mean ± standard deviation (SD), and statistical analysis was performed using the student's *t*-test or one-way analysis of variance (ANOVA) by SPSS software. *P* value <0.05 was seen as significant differences.

## 3. Results

### 3.1. Potential Bioactive Components of YHPGKL and Possible Target Proteins

**Figure 1:**
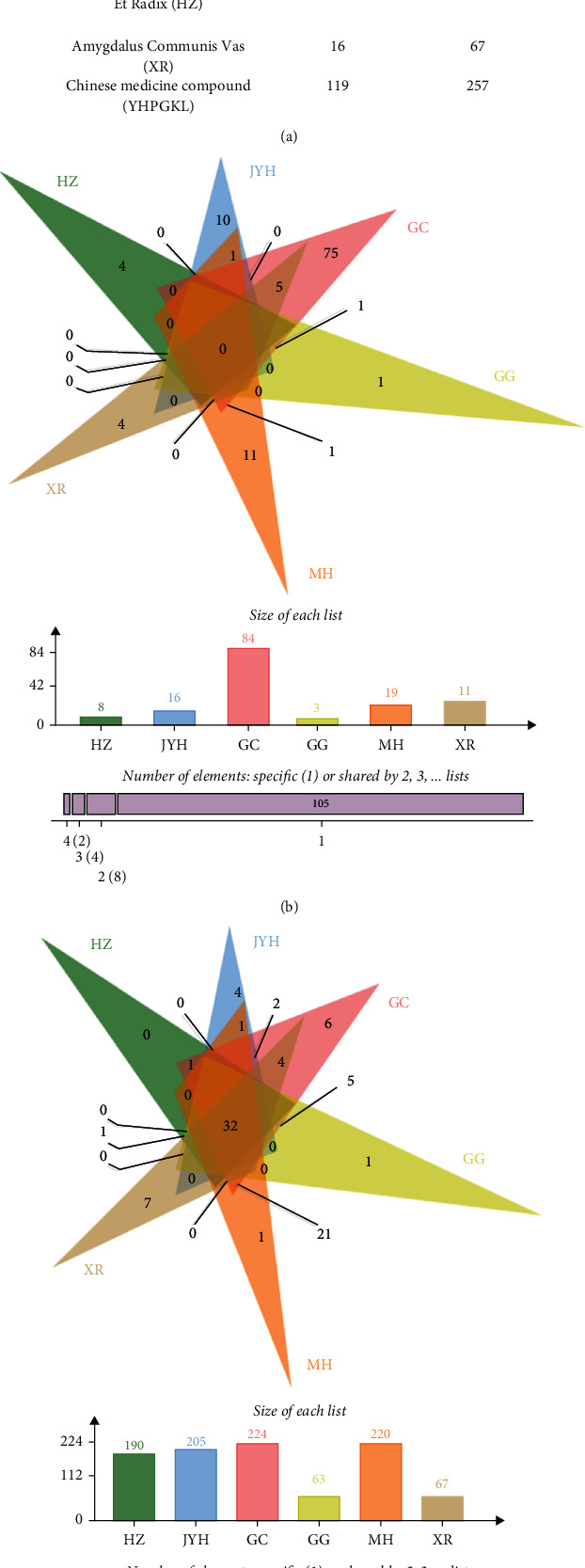
The bioactive components of YHPGKL and target proteins. (a) The numbers of bioactive components and target proteins of herbs and the YHPGKL were shown. The distribution of bioactive components (b) and target proteins (c) of YHPGKL were analyzed by Venn diagram.

According to the standard of OB >30% and DL >0.18, which has been performed by other studies [11, 37], 119 bioactive components of YHPGKL were identified based on TCMSP, of which 17 were in JYH, 88 in GC, 4 in GG, 22 in MH, 10 in HZ, and 16 in XR (Figure 1(a)). In addition, a total of 257 target proteins of YHPGKL were affirmed based on the Drugbank database, of which 205 were targeted by JYH, 230 by GC, 63 by GG, 220 by MH, 190 by HZ, and 67 by XR (Figure 1(a)). By means of Venn diagram, these bioactive components were distributed into six herbs, but some were in more than one herb (Figure 1(b)). A similar result was also found by the distribution analysis of target proteins of YHPGKL (Figure 1(c)).

### 3.2. Potential Effective Components of YHPGKL and Common Target Proteins with Pneumonia

**Figure 2:**
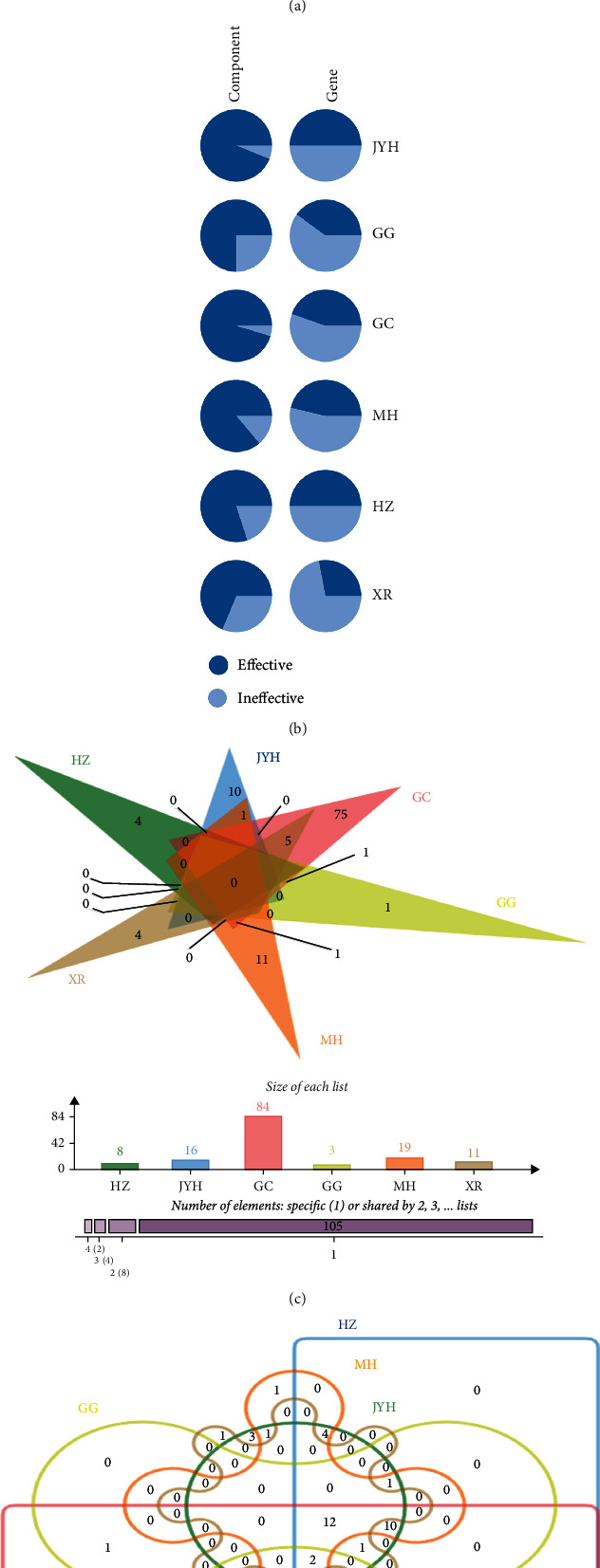
Analysis of common targets of YHPGKL and pneumonia. (a) The common targets between YHPGKL and pneumonia were screened by Venn diagram. (b) The proportion of effective components and genes in each herb was shown. The distribution of effective components (c) and common targets (d) of each herb was visualized by Venn diagram.

Due to pneumonia's complex mechanisms and bioprocesses, we screened GenCards (score> 3) and the OMIN database on account of similar studies and obtained 1423 pneumonia-related targets [37, 38]. Then, 113 common targets between YHPGKL and pneumonia were identified by Venn analysis (Figure 2(a)). Next, we analyzed the proportion of effective components vs. ineffective components (rest bioactive components) and effective genes (common targets) vs. ineffective genes (rest targets) in each herb (Figure 2(b)). The results demonstrated that each herb's effective components and target proteins are over 50%. Furthermore, by distribution analysis, we found 117 effective components of YHPGKL, among which 8 were from HZ, 16 from JYH, 84 from GC, 3 from GG, 19 from MH, and 11 from XR, and some of which were in more than one herb (Figure 2(c)). Similarly, 113 common targets were in more than one herb (Figure 2(d)).

### 3.3. Compound—Effective Component—Target Protein Network and Potential Hub Effective Components

**Figure 3:**
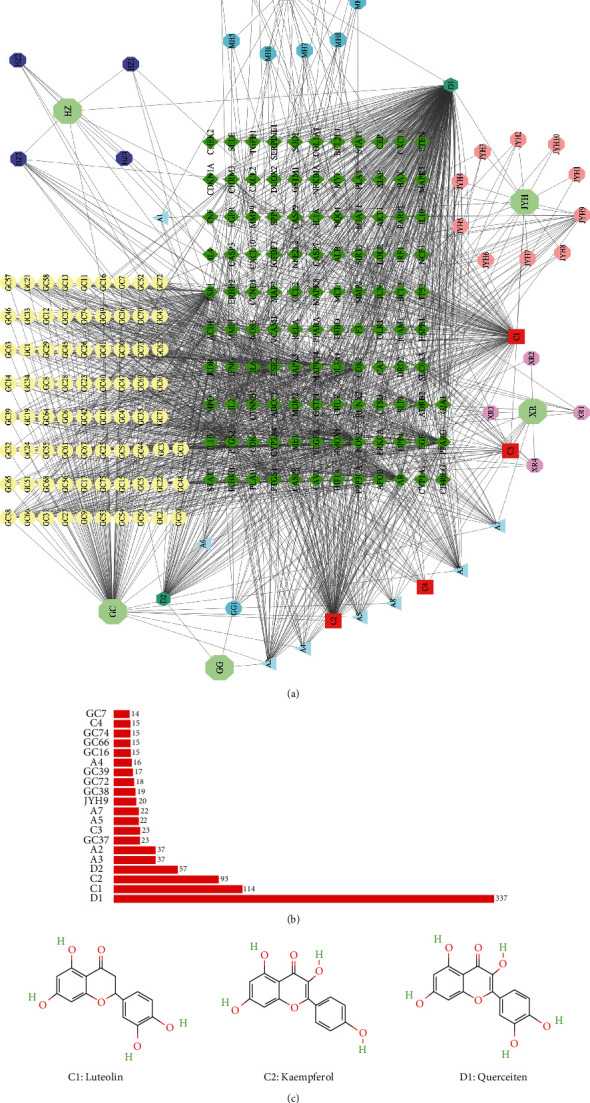
Compound—effective component—target protein network establishment and hub components analysis. (a) The Compound—effective component—target protein network was built. The degrees were analyzed (b) and the top three hub components were screened out (c).

Based on the above results, we set up a network to visualize the interactions of 6 herbs, effective components, and target proteins by Cytoscape (Figure 3(a) and Table. S1). The degree value identified three effective hub components with degrees >90, namely luteolin, kaempferol, and quercetin (Figures 3(b) and 3(c)).

### 3.4. PPI Network and Enrichment Analysis

**Figure 4:**
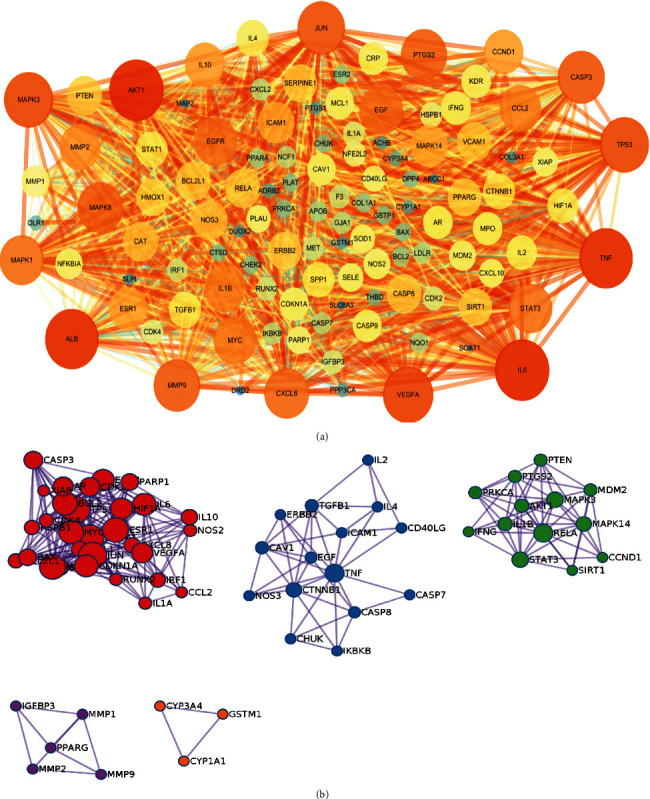
PPI analysis for common target proteins. PPI analyzed the 116 common targets (a) and MCODE analysis was further performed by Metascape (b).

**Figure 5:**
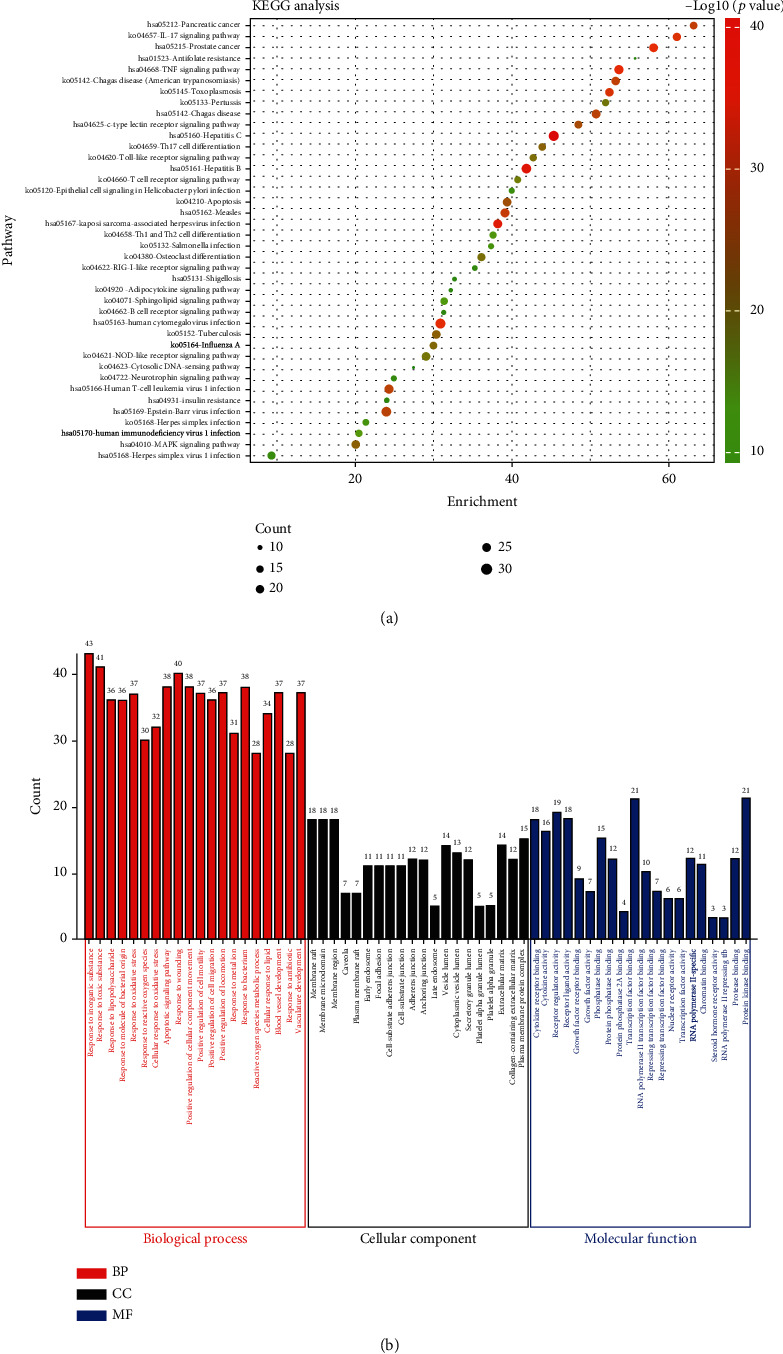
KEGG and GO analysis. The 116 common target proteins were analyzed by KEGG (a) and GO (b) analysis.

To elucidate the mechanisms of YHPGKL in the treatment of pneumonia, we refereed to values of similar studies, and firstly used the STRING database and Cytoscape to perform the protein-protein interaction of 116 common targets proteins [11, 37]. As shown in Figure 4(a), the bigger and more profound the node (protein) is, the higher its degree is, and thus the more important it may be. Then, the MCODE was used by Metascape to cluster the PPI network, and as shown in Figure 4(b), five clusters were obtained. To further explore the bioprocess and signaling pathway of YHPGKL to pneumonia, we used Metascape to visualize KEGG and GO functional enrichment analysis on account of 113 common targets proteins (Figure 5). As shown in Figure 5(a), we selected the top 40 significant terms in KEGG in line with *P* value. In addition, the biological process (BP), cellular components (CC), and Molecular function (MF) were analyzed, and the top 20 terms were performed, respectively (Figure 5(b)). Besides, the BP, CC, and MF cluster analyses were also visualized by Metascape, respectively (Figure S1). Results noted that most signaling pathways were related to inflammatory response, pathogen infection, and immune reaction.

### 3.5. Potential Hub Target Proteins and Herb–Hub Effective Component–Hub Target Protein Network

**Figure 6:**
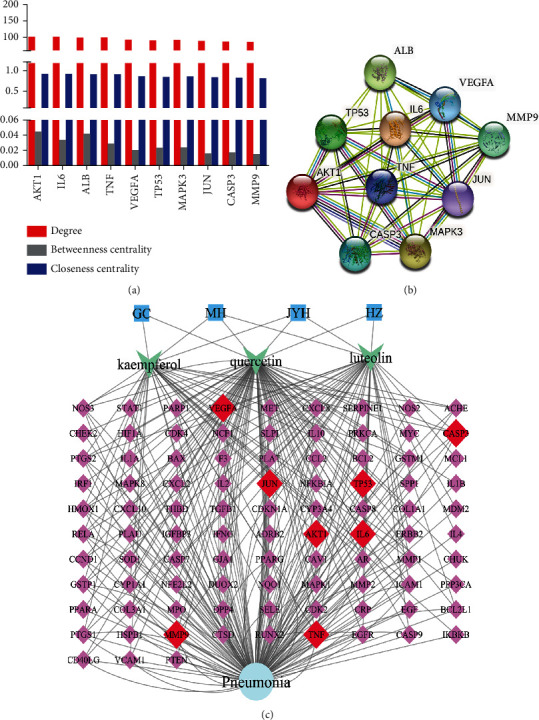
The effective hub component-hub protein network establishment. (a) The top ten hub proteins were screened out by PPI analysis. (b) The hub proteins were analyzed by PPI. Edges represent protein-protein associations. Sky-blue edge bases on curated databases; purple edge bases on experimentally determined; green edge bases on gene neighborhood; Red edge bases on gene fusions; blue edge bases on gene CP occurrence; yellow edge bases on text mining; black edge bases on coexpression; and violet edge bases on protein homology. (c) The effective hub component-target protein network was built, and hub proteins were shown in the red node.

As shown in Figure 6(c), we set up an herb-effective hub component-common target protein-pneumonia network based on effective hub components related to common target proteins. Based on Figure 4(a), the ten hub target proteins were screened out by degrees, namely AKT1, IL6, ALB, TNF, VEGF, TP53, MAPK3, JUN, CASP3, and MMP9 (Figure 6(a)). The PPI network of 10 hub target proteins has 45 edges, 10 nodes, 30 expected number of edges, avg. Local clustering coefficient = 1, an average node degree = 9, and *P* value = 0.0108 (Figure 6(b)) and detailed information was shown in Table S2. Only 8 hub target proteins (red node) can interact with three effective hub components. Thus, the 3 hub effective components (luteolin, kaempferol, and quercetin) and 8 targets (VEGF, CASP3, JUN, TP53, AKT1, IL6, TNF, and MMP9) might play essential roles in the treatment of pneumonia by YHPGKL.

### 3.6. Validation with Molecular Docking and Hub Effective Component–Hub Target Protein–Signaling Pathway Network

**Figure 7:**
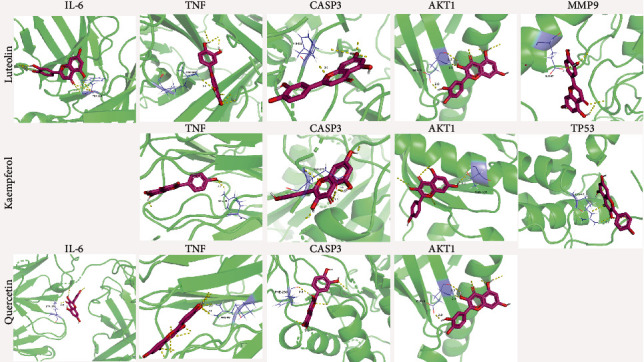
Molecular docking analysis. Three components interacted with eight proteins, respectively. Only binding energy> -4,25 kcal/moL of component-protein was shown.

The eight hub target proteins and three effective hub components were used as ligands and receptors by docking analysis. Because of the ligand and receptor binding stability depending on the binding energy, with lower binding energy resulting in more stable binding, therefore, the binding energy -4.25 kcal/moL was set as a threshold in the present study [37, 39]. As shown in Figure 7, luteolin could stably bind with IL6 (-4.83 kcal/moL), TNF (-6.16 kcal/moL), CASP3 (-7 kcal/moL), AKT1 (-4.88 kcal/moL), and MMP9 (-4.67 kcal/moL), while kaempferol bound to TNF (-5.48 kcal/moL), CASP3 (-5.05 kcal/moL), AKT1 (-4.72 kcal/moL), and TP53(-4.29 kcal/moL), as quercetin bound to IL6 (-4.51 kcal/moL), TNF (-5.83 kcal/moL), AKT1 (-4.88 kcal/moL), and CASP3 (-4.29 kcal/moL).

**Figure 8:**
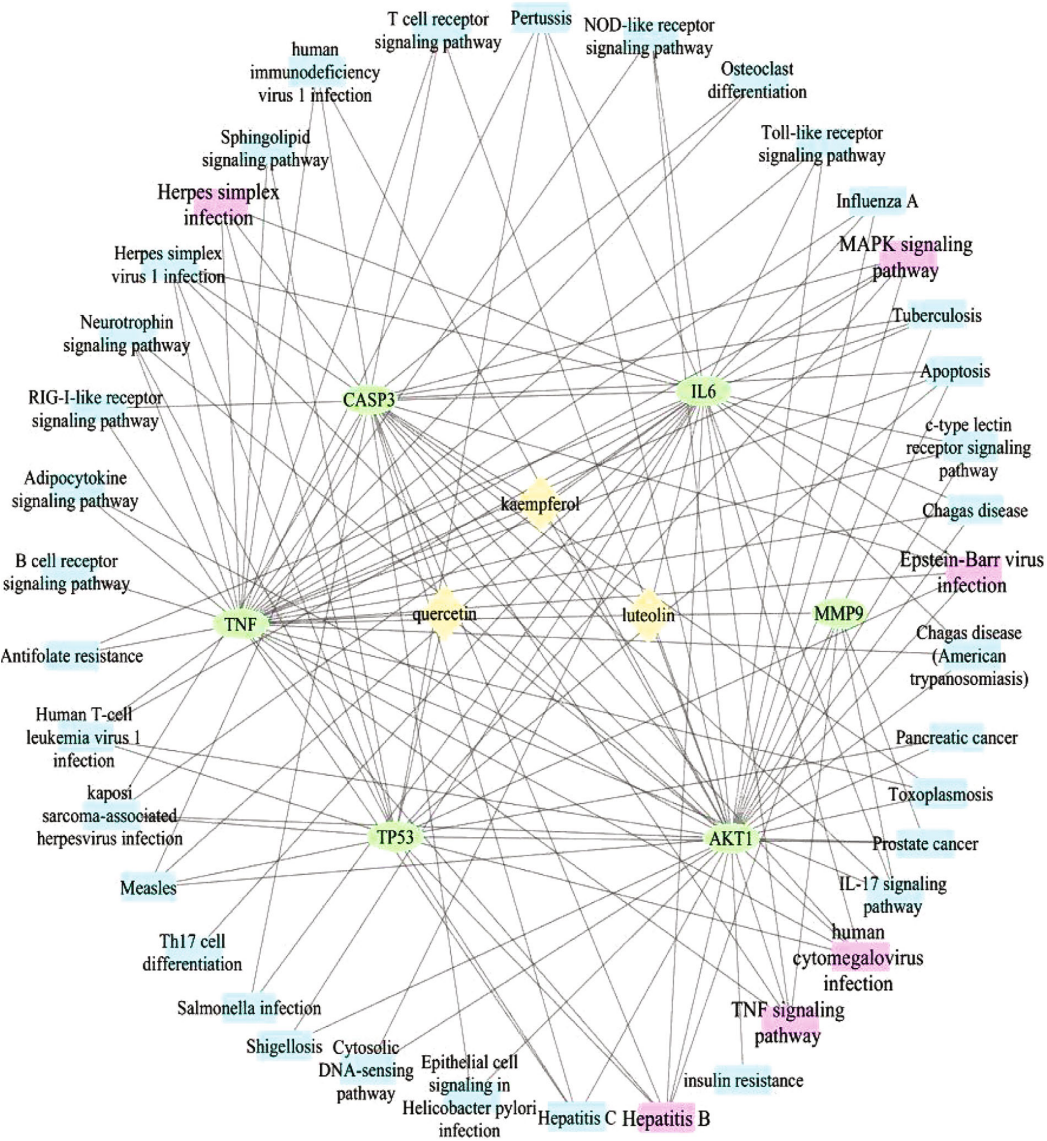
Effective hub component-hub proteins-signaling pathways network. The effective hub component-hub proteins-related signaling pathways network was built, among which hub signaling pathways were marked purple with degrees >4. The signaling pathways by KEGG analysis were further screened out based on the distribution of 6 hub proteins.

We finally set up an effective hub component–hub target protein–signaling pathway network with Cytoscape based on the above results for exploring potential central signaling pathways. Our docking results demonstrated that the hub components could interact with hub target proteins. As shown in Figure 8, we screened out six major signaling pathways with degrees (>4), which might play important roles during the interaction between hub components and hub target proteins. Of note, most signaling pathways are relative to the immune system, especially in inflammation, suggesting these hub effective components functioning in immune cells in the treatment of pneumonia.

### 3.7. *In Vitro* Study for the Interaction of Hub Effective Components and Hub Target Proteins

**Figure 9:**
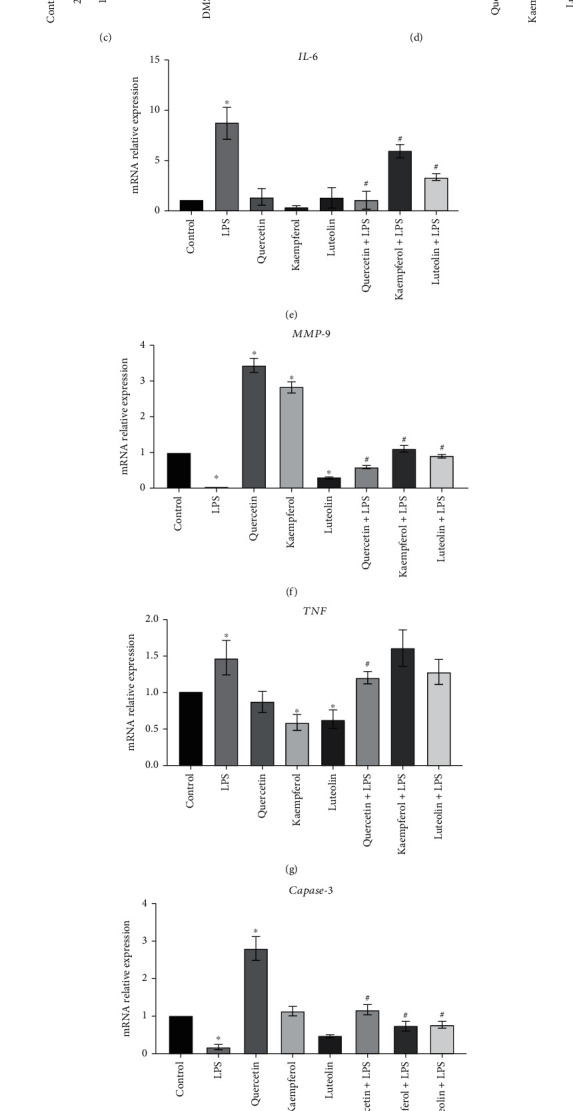
Analysis of effective hub components and hub genes *in vitro*. (ac) The cell viability of macrophages was analyzed after incubation with luteolin, kaempferol, or quercetin at various concentrations for 24 h. RT-qPCR analyzed for TP53 (d), IL-6 (e), MMP-9 (f), TNF (g), Caspase-3 (h), and AKT1 (i) expressions of macrophage after incubation in the presence of luteolin, kaempferol, or quercetin with or without 100 ng/ml LPS stimulation for 24 h. ^∗^*P* < 0.05 vs. control, ^#^*P* < 0.05 vs. LPS.

In terms of the above network results, we next analyzed the relationships between three hub effective components and six target proteins. Due to the function of these components focusing on immune cells (Figure 8), we herein preformed macrophage, an innate immune cell generally seen as a first defense during infection and an inflammatory trigger, as a cell model and also establish an inflammatory cell model of by using LPS, a major component of cell walls of gram-negative bacteria to mimic lung injury [40, 41]. Firstly, we determined the cytotoxicity of macrophages at various concentrations of luteolin, kaempferol, and quercetin, respectively. As shown in Figures 9(a)–9(c), luteolin, kaempferol, and quercetin showed cytotoxicity at >50 *μ*M, 25 *μ*M, and40*μ*M, respectively. Thus, we selected concentrations of 25 *μ*M, 12.5 *μ*M, and 20 *μ*M of these effective components in the following experiments. Next, we assayed the changes of targeted genes identified by molecular docking during which macrophages were added luteolin, kaempferol, or quercetin with or without LPS stimulation. As shown in Figure 9(d)–9(i), luteolin, kaempferol, and quercetin were, respectively, inhibited proinflammatory genes (IL6 and TNF) expression during LPS stimulation, though kaempferol and quercetin could upregulate MMP-9 expression. Of note, luteolin and kaempferol attenuated TNF expression without LPS stimulation. As for cell survival genes (AKT1, Caspase-3, and TP53), quercetin could lonely upregulate Caspase-3 expression, but both three components with LPS increased Caspase-3 expression compared with LPS alone group. Interestingly, quercetin combined with LPS significantly increased TP53 expression compared to LPS alone. Thus, these results demonstrated that luteolin, kaempferol, or quercetin could interact with IL6, TNF, and MMP-9 instead of AKT1, Caspase-3, and TP53.

## 4. Discussion

The abuse of antibiotics and other medicines to treat pneumonia gives rise to multiple problems, including organ damage, allergy, and refractory infection. TCM has a long history of clinical application based on its unique theory and combination of multiherbs 10. YHPGKL is an improved TCM compound found on MHT, and in our previous study, encouraging results emerged in the therapy of various pneumonia, influenza, etc. [15, 21, 22] Due to multiple components, multiple targets, and complex pathways, the precise mechanisms of YHPGKL are needed to be investigated. This study analyzed and screened the potential effective hub components, hub targets, and central signaling pathways of YHPGKL to treat pneumonia using network pharmacology and *in vitro*.

The OB value and DL were set as a standard in the TCMSP database to ensure that those components functioned *in vivo* [39]. We screened out the top three effective components, namely luteolin, kaempferol, and quercetin, from the YHPGKL, which might significantly affect pneumonia. Previous data found that luteolin had an anti-inflammatory capacity by inhibiting NF-*κ*B activity, reducing interleukin (IL) 6, tumor necrosis factor (TNF) -*α*, etc., expression [42]. In addition, it has been reported that luteolin can inhibit apoptosis by activating Akt/Nrf2/HO-1 pathway [43]. Our study's target prediction, docking analysis network, and *in vitro* experiment suggested interacting with IL6, MMP-9, Caspase-3, and TNF, consistent with the previous report. Of note, the docking analysis demonstrated that a structural core interaction between compound and critical protein, which deserve to further study with such as increase binding activity by chemical modification, etc.

Growing evidence has proved that kaempferol has an excellent protective ability against inflammation and apoptosis *in vivo* and *in vitro* [44, 45]. Mechanically, Chen et al. reported that kaempferol attenuated the MAPK and NF-*κ*B pathway activity and inhibited the expression of proinflammatory cytokines in the lung tissue of LPS induced ALI mice model [46]. Du et al. showed that kaempferol inhibited cell damage and apoptosis via regulating Caspase-3 and P53 molecules [47]. Following other results, our study confirmed it could bind to TNF and Caspase-3. Quercetin has been widely reported to have anti-inflammatory activities and antioxidants [48]. Our results suggested that quercetin can interact with some proteins involved in IL6, TNF, MMP-9, and Caspase-3. Together, the three components could regulate anti-inflammatory, playing critical roles in pneumonia and implying their central function in the YHPGKL.

KEGG and GO analysis reveal these common targets distribution in all signaling pathways, biological processes, cellular components, and molecular function. In our studies, the top ranks of signaling pathway are involved in immune-related signaling pathways, such as the TNF pathway, IL-17pathway, Toll-like pathway, and MAPK pathway. Besides, the top ranks of biological process are response to exogenous invasion, oxidative stress, and cell injury, like molecular of bacterial origin, oxidative stress and wounding, etc. Furthermore, the top ranks of molecular function focus on inflammatory response (e.g. cytokines receptor binding, cytokine activity, growth factor activity, etc.). Also, the top ranks of cellular component revealed cell mobilization and stress such as membrane raft, early endosome and focal adhesion, etc. In terms of KEGG and GO results, these effective components of YHPGKL are obviously interacted with pneumonia through regulating inflammation and wounding.

Further study, the top three effective components from YHPGKL were picked up and also tightly related with inflammatory genes (Figures 4–6). According to molecular docking results, we found the three components could be interacted with TNF and Caspase-3, and critical downstream protein as well. Furthermore, the Rt-qPCR result also showed these components significantly regulated TNF and Caspase-3 in cells in the different conditions. Therefore, based on these findings, we speculate that the target proteins by the three hub components are involved in TNF signaling pathway, which plays possible regulation amid YHPGKL against pneumonia.

In fact, it has been reported that TNF signaling pathway serves a vital role in a broad spectrum of diseases [49]. TNF is produced by various immune cells such as monocyte/macrophage, T and B lymphocytes, and NK cells. [50]. By binding of TNF receptor, TNFR1or TNFR2, on the cell surface regulated several cellular functions implicating programmed cell death, proliferation, tissue regeneration, or inflammation. For example, TNFR1 bound by TNF leads to apoptosis through transduction signals to the caspase proteins [49]. Intriguingly, in our recent report, the YHPGKL could play antiapoptosis during pneumonia, and this study also found that the effective hub components enable interaction with the TNF pathway [23]. So, it is acceptable to define the TNF signaling pathway of pneumonia as a critical therapeutic target of YHPGKL, which is necessary to analyze in the future.

In a nut word, the network pharmacology help us analyze possible effective components of YHPGKL, illuminate the potential mechanisms of compounds, improve compounds, and extract valuable components from YHPGKL, all of which could promote experimental processes in real-world experiments. However, it is worth noting that the herb compound showed extremely complex metabolism *in vivo*, resulting in variable active components, hardly predicting actual effective components and contents. Therefore, we performed *in vitro* experiment to confirm the above results. The discrepancy between network pharmacology and *in vitro* was acceptable due to virtual and real-world research. In addition, although the top three compounds we screened out play important roles in the treatment of pneumonia, according to our studies, they either interacted with common targets or different targets, suggesting a possible combination (i.e., “cocktail”) and is worth to finding an appropriate matching in the future.

## 5. Conclusions

In this research, we systemically analyzed the potential effective components and target proteins of YHPGKL in the treatment of pneumonia through network pharmacology, molecular docking analysis, and *in vitro*. We screened out three hub-effective components and six critical proteins, possibly playing pivotal roles in protecting against lung injury. Importantly, our results implied that the TNF signaling pathway might be a potential mechanism by which YHPGKL treats pneumonia, which will be confirmed.

## Data Availability

All supporting data are included within the main article and its supplementary files.

## Supplementary Materials

Supplementary Materials Supplementary Figure legends Supplementary Figure 1: the cluster analysis of GO. the biological process (a), cellular components (b), and molecular function (c) were analyzed by cluster analysis, respectively. Supplementary Table 1 Title: the chemical name of effective components (as noted in alphabets) in Figure 3(a). Supplementary Table 2 Title: the PPI analysis.
