# Supplementary material for: Investigation of Pharmacological Mechanisms of Yinhua Pinggan Granule on the Treatment of Pneumonia through Network Pharmacology and In Vitro
Source: Biomed Res Int. 2022 Nov 2;2022:1602447. doi: 10.1155/2022/1602447 (PMC9646329; doi:10.1155/2022/1602447)
Supplement: Supplementary Materials — Supplementary Figure legends Supplementary Figure 1: the cluster analysis of GO. the biological process (a), cellular components (b), and molecular function (c) were analyzed by cluster analysis, respectively. Supplementary Table 1 Title: the chemical name of effective components (as noted in alphabets) in Figure 3(a). Supplementary Table 2 Title: the PPI analysis. [file 1602447.f1.zip › Table S1.docx]

Title: the chemical name of effective components (as noted in alphabets) in the Fig, 3A.

| Serial Number | Component | Herb Name |
| --- | --- | --- |
| HZ1  HZ2  HZ3  HZ4 | [Rhein](https://tcmspw.com/molecule.php?qn=2268)  [6,8-Dihydroxy-7-methoxyxanthone](https://tcmspw.com/molecule.php?qn=13281)  [Physovenine](https://tcmspw.com/molecule.php?qn=13287)  [Picralinal](https://tcmspw.com/molecule.php?qn=13288) | HZ |
| JYH1  JYH2  JYH3  JYH4  JYH5  JYH6  JYH7  JYH8  JYH9  JYH10 | Dinethylsecologanoside  Ioniceracetalides B_qt  Centauroside_qt  5-hydroxy-7-methoxy-2-(3,4,5-trimethoxyphenyl)chromone  Chryseriol  Secologanic dibutylacetal_qt  -)-(3R,8S,9R,9aS,10aS)-9-ethenyl-8-(beta-D-glucopyranosyloxy)-2,3,9,9a,10,10a-hexahydro-5-oxo-5H,8H-pyrano[4,3-d]oxazolo[3,2-a]pyridine-3-carboxylic acid_qt  Eriodyctiol (flavanone)  Beta-carotene  Ethyl linolenate | JYH |
| GC1  GC2  GC3  GC4  GC5  GC6  GC7  GC8  GC9  GC10  GC11  GC12  GC13  GC14  GC15  GC16  GC17  GC18  GC19  GC20  GC21  GC22  GC23  GC24  GC25  GC26  GC27  GC28  GC29  GC30  GC31  GC32  GC33  GC34  GC35  GC36  GC37  GC38  GC39  GC40  GC41  GC42  GC43  GC44  GC45  GC46  GC47  GC48  GC49  GC50  GC51  GC52  GC53  GC54  GC55  GC56  GC57  GC58  GC59  GC60  GC61  GC62  GC63  GC64  GC65  GC66  GC67  GC68  GC69  GC70  GC71  GC72  GC73  GC74  GC75 | Dehydroglyasperins C  Xambioona  Odoratin  Licoagroisoflavone  Glycyrrhiza flavonol A  Glyasperins M  Licoagrocarpin  Gancaonin H  Gancaonin G  8-prenylated eriodictyol  7-Acetoxy-2-methylisoflavone  7,2',4'-trihydroxy－5-methoxy-3－arylcoumarin  6-prenylated eriodictyol  Kanzonol F  Inflacoumarin A  2-[(3R)-8,8-dimethyl-3,4-dihydro-2H-pyrano[6,5-f]chromen-3-yl]-5-methoxyphenol  3'-Methoxyglabridin  3'-Hydroxy-4'-O-Methylglabridin  Quercetin der.  1-Methoxyphaseollidin  HMO  Isolicoflavonol  licochalcone G  (2S)-7-hydroxy-2-(4-hydroxyphenyl)-8-(3-methylbut-2-enyl)chroman-4-one  (2R)-7-hydroxy-2-(4-hydroxyphenyl)chroman-4-one  Sigmoidin-B  (-)-Medicocarpin  Eurycarpin A  1,3-dihydroxy-8,9-dimethoxy-6-benzofurano[3,2-c]chromenone  1,3-dihydroxy-9-methoxy-6-benzofurano[3,2-c]chromenone  Glabrone  Glabrene  Glabranin  Glyzaglabrin  Licopyranocoumarin  (E)-3-[3,4-dihydroxy-5-(3-methylbut-2-enyl)phenyl]-1-(2,4-dihydroxyphenyl)prop-2-en-1-one  Shinpterocarpin  Licoisoflavanone  Licoisoflavone B  Licoisoflavone  Licocoumarone  Glycyrin  2-(3,4-dihydroxyphenyl)-5,7-dihydroxy-6-(3-methylbut-2-enyl)chromone  5,7-dihydroxy-3-(4-methoxyphenyl)-8-(3-methylbut-2-enyl)chromone  3-(3,4-dihydroxyphenyl)-5,7-dihydroxy-8-(3-methylbut-2-enyl)chromone  Gancaonin B  Gancaonin A  Licoricone  3-(2,4-dihydroxyphenyl)-8-(1,1-dimethylprop-2-enyl)-7-hydroxy-5-methoxy-coumarin  Licochalcone G  8-(6-hydroxy-2-benzofuranyl)-2,2-dimethyl-5-chromenol  Glypallichalcone  Phaseolinisoflavan  Glepidotin B  Glepidotin A  Semilicoisoflavone B  (2S)-6-(2,4-dihydroxyphenyl)-2-(2-hydroxypropan-2-yl)-4-methoxy-2,3-dihydrofuro[3,2-g]chromen-7-one  Kanzonols W  (E)-1-(2,4-dihydroxyphenyl)-3-(2,2-dimethylchromen-6-yl)prop-2-en-1-one  Isotrifoliol  Glyasperin C  Glyasperin F  Glyasperin B  Euchrenone  (2S)-2-[4-hydroxy-3-(3-methylbut-2-enyl)phenyl]-8,8-dimethyl-2,3-dihydropyrano[2,3-f]chromen-4-one  7-Methoxy-2-methyl isoflavone  Lupiwighteone  Medicarpin  DFV  Inermine  Vestitol  licochalcone a  Calycosin  Isorhamnetin  Jaranol | GC |
| GG1 | 3'-Methoxydaidzein | GG |
| MH1  MH2  MH3  MH4  MH5  MH6  MH7  MH8  MH9  MH10  MH11 | Leucopelargonidin  Herbacetin  Resivit  Delphinidin  Diosmetin  Taxifolin  Eriodictyol  Genkwanin  Pectolinarigenin  (+)-Leucocyanidin  Truflex OBP | MH |
| XR1  XR2  XR3  XR4 | Estrone  Gondoic acid  Machiline  l-SPD | XR |
| A1 | Mandenol | JYH,MH |
| A2 | [Formononetin](https://tcmspw.com/molecule.php?qn=392) | GC,GG |
| A3 | [Naringenin](https://tcmspw.com/molecule.php?qn=4328) | MH,GC |
| A4 | [Phaseol](https://tcmspw.com/molecule.php?qn=5017) | GC,XR |
| A5 | [Glabridin](https://tcmspw.com/molecule.php?qn=4908) | GC,XR |
| A6 | [Liquiritin](https://tcmspw.com/molecule.php?qn=4903) | GC,XR |
| A7 | [Licochalcone B](https://tcmspw.com/molecule.php?qn=4841) | GC,XR |
| A8 | [Glycyrol](https://tcmspw.com/molecule.php?qn=2311) | GC,XR |
| C1 | [Luteolin](https://tcmspw.com/molecule.php?qn=6) | JYH,MH,XR |
| C2 | [Kaempferol](https://tcmspw.com/molecule.php?qn=422) | JYH,MH,GC |
| C3 | [Stigmasterol](https://tcmspw.com/molecule.php?qn=449) | JYH,MH,XR |
| C4 | [(+)-catechin](https://tcmspw.com/molecule.php?qn=492) | MH,HZ,XR |
| D1 | [Quercetin](https://tcmspw.com/molecule.php?qn=98) | JYH,HZ,GC,MH |
| D2 | [Beta-sitosterol](https://tcmspw.com/molecule.php?qn=358) | JYH,GG,MH,HZ |
