# Supplementary material for: Investigation of Pharmacological Mechanisms of Yinhua Pinggan Granule on the Treatment of Pneumonia through Network Pharmacology and In Vitro
Source: Biomed Res Int. 2022 Nov 2;2022:1602447. doi: 10.1155/2022/1602447 (PMC9646329; doi:10.1155/2022/1602447)
Supplement: Supplementary Materials — Supplementary Figure legends Supplementary Figure 1: the cluster analysis of GO. the biological process (a), cellular components (b), and molecular function (c) were analyzed by cluster analysis, respectively. Supplementary Table 1 Title: the chemical name of effective components (as noted in alphabets) in Figure 3(a). Supplementary Table 2 Title: the PPI analysis. [file 1602447.f1.zip › Table S2.docx]

The PPI analysis

| #node1 | node2 | node1_string_id | node2_string_id | neighborhood_on_chromosome | gene_fusion | phylogenetic_cooccurrence | homology | coexpression | experimentally_determined_interaction | database_annotated | automated_textmining | combined_score |
| --- | --- | --- | --- | --- | --- | --- | --- | --- | --- | --- | --- | --- |
| AKT1 | TIMP1 | 9606.ENSP00000451828 | 9606.ENSP00000218388 | 0 | 0 | 0 | 0 | 0 | 0.058 | 0 | 0.603 | 0.61 |
| AKT1 | MAPK3 | 9606.ENSP00000451828 | 9606.ENSP00000263025 | 0 | 0 | 0.323 | 0.586 | 0.066 | 0.148 | 0.8 | 0.938 | 0.906 |
| AKT1 | TP53 | 9606.ENSP00000451828 | 9606.ENSP00000269305 | 0 | 0 | 0 | 0 | 0.053 | 0.201 | 0.9 | 0.877 | 0.989 |
| AKT1 | ALB | 9606.ENSP00000451828 | 9606.ENSP00000295897 | 0 | 0 | 0 | 0 | 0 | 0 | 0 | 0.792 | 0.792 |
| AKT1 | CASP3 | 9606.ENSP00000451828 | 9606.ENSP00000311032 | 0 | 0 | 0 | 0 | 0 | 0.475 | 0 | 0.882 | 0.935 |
| AKT1 | JUN | 9606.ENSP00000451828 | 9606.ENSP00000360266 | 0 | 0 | 0 | 0 | 0 | 0.057 | 0.8 | 0.877 | 0.974 |
| AKT1 | IL6 | 9606.ENSP00000451828 | 9606.ENSP00000385675 | 0 | 0 | 0 | 0 | 0.056 | 0 | 0 | 0.829 | 0.832 |
| AKT1 | TNF | 9606.ENSP00000451828 | 9606.ENSP00000398698 | 0 | 0 | 0 | 0 | 0 | 0.056 | 0 | 0.826 | 0.829 |
| AKT1 | VEGFA | 9606.ENSP00000451828 | 9606.ENSP00000478570 | 0 | 0 | 0 | 0 | 0.063 | 0.213 | 0 | 0.871 | 0.896 |
| ALB | TIMP1 | 9606.ENSP00000295897 | 9606.ENSP00000218388 | 0 | 0 | 0 | 0 | 0 | 0 | 0 | 0.606 | 0.606 |
| ALB | MAPK3 | 9606.ENSP00000295897 | 9606.ENSP00000263025 | 0 | 0 | 0 | 0 | 0 | 0 | 0 | 0.702 | 0.702 |
| ALB | TP53 | 9606.ENSP00000295897 | 9606.ENSP00000269305 | 0 | 0 | 0 | 0 | 0 | 0 | 0 | 0.715 | 0.715 |
| ALB | JUN | 9606.ENSP00000295897 | 9606.ENSP00000360266 | 0 | 0 | 0 | 0 | 0 | 0 | 0 | 0.698 | 0.698 |
| ALB | VEGFA | 9606.ENSP00000295897 | 9606.ENSP00000478570 | 0 | 0 | 0 | 0 | 0.062 | 0 | 0 | 0.78 | 0.785 |
| ALB | CASP3 | 9606.ENSP00000295897 | 9606.ENSP00000311032 | 0 | 0 | 0 | 0 | 0 | 0 | 0 | 0.852 | 0.852 |
| ALB | IL6 | 9606.ENSP00000295897 | 9606.ENSP00000385675 | 0 | 0 | 0 | 0 | 0 | 0 | 0 | 0.86 | 0.86 |
| ALB | TNF | 9606.ENSP00000295897 | 9606.ENSP00000398698 | 0 | 0 | 0 | 0 | 0 | 0 | 0 | 0.866 | 0.866 |
| CASP3 | TIMP1 | 9606.ENSP00000311032 | 9606.ENSP00000218388 | 0 | 0 | 0 | 0 | 0 | 0 | 0 | 0.558 | 0.558 |
| CASP3 | MAPK3 | 9606.ENSP00000311032 | 9606.ENSP00000263025 | 0 | 0 | 0 | 0 | 0 | 0.057 | 0.8 | 0.821 | 0.963 |
| CASP3 | TP53 | 9606.ENSP00000311032 | 9606.ENSP00000269305 | 0 | 0 | 0 | 0 | 0.062 | 0.27 | 0 | 0.9 | 0.925 |
| CASP3 | VEGFA | 9606.ENSP00000311032 | 9606.ENSP00000478570 | 0 | 0 | 0 | 0 | 0 | 0 | 0 | 0.76 | 0.76 |
| CASP3 | IL6 | 9606.ENSP00000311032 | 9606.ENSP00000385675 | 0 | 0 | 0 | 0 | 0 | 0 | 0 | 0.836 | 0.836 |
| CASP3 | JUN | 9606.ENSP00000311032 | 9606.ENSP00000360266 | 0 | 0 | 0 | 0 | 0 | 0.057 | 0 | 0.834 | 0.837 |
| CASP3 | TNF | 9606.ENSP00000311032 | 9606.ENSP00000398698 | 0 | 0 | 0 | 0 | 0 | 0.102 | 0 | 0.889 | 0.896 |
| IL6 | TIMP1 | 9606.ENSP00000385675 | 9606.ENSP00000218388 | 0 | 0 | 0 | 0 | 0.1 | 0 | 0.9 | 0.74 | 0.974 |
| IL6 | MAPK3 | 9606.ENSP00000385675 | 9606.ENSP00000263025 | 0 | 0 | 0 | 0 | 0 | 0 | 0.9 | 0.741 | 0.973 |
| IL6 | TP53 | 9606.ENSP00000385675 | 9606.ENSP00000269305 | 0 | 0 | 0 | 0 | 0.062 | 0 | 0 | 0.738 | 0.744 |
| IL6 | JUN | 9606.ENSP00000385675 | 9606.ENSP00000360266 | 0 | 0 | 0 | 0 | 0.052 | 0 | 0.9 | 0.822 | 0.981 |
| IL6 | VEGFA | 9606.ENSP00000385675 | 9606.ENSP00000478570 | 0 | 0 | 0 | 0 | 0.063 | 0 | 0.9 | 0.879 | 0.987 |
| IL6 | TNF | 9606.ENSP00000385675 | 9606.ENSP00000398698 | 0 | 0 | 0 | 0 | 0.125 | 0 | 0.9 | 0.941 | 0.994 |
| JUN | TIMP1 | 9606.ENSP00000360266 | 9606.ENSP00000218388 | 0 | 0 | 0 | 0 | 0 | 0 | 0 | 0.557 | 0.557 |
| JUN | MAPK3 | 9606.ENSP00000360266 | 9606.ENSP00000263025 | 0 | 0 | 0 | 0 | 0 | 0.5 | 0.9 | 0.852 | 0.991 |
| JUN | TP53 | 9606.ENSP00000360266 | 9606.ENSP00000269305 | 0 | 0 | 0 | 0 | 0 | 0.149 | 0.6 | 0.838 | 0.94 |
| JUN | VEGFA | 9606.ENSP00000360266 | 9606.ENSP00000478570 | 0 | 0 | 0 | 0 | 0.066 | 0.182 | 0.9 | 0.683 | 0.972 |
| JUN | TNF | 9606.ENSP00000360266 | 9606.ENSP00000398698 | 0 | 0 | 0 | 0 | 0 | 0 | 0.9 | 0.847 | 0.984 |
| MAPK3 | TIMP1 | 9606.ENSP00000263025 | 9606.ENSP00000218388 | 0 | 0 | 0 | 0 | 0 | 0 | 0 | 0.512 | 0.512 |
| MAPK3 | VEGFA | 9606.ENSP00000263025 | 9606.ENSP00000478570 | 0 | 0 | 0 | 0 | 0 | 0 | 0 | 0.754 | 0.754 |
| MAPK3 | TNF | 9606.ENSP00000263025 | 9606.ENSP00000398698 | 0 | 0 | 0 | 0 | 0 | 0 | 0 | 0.765 | 0.765 |
| MAPK3 | TP53 | 9606.ENSP00000263025 | 9606.ENSP00000269305 | 0 | 0 | 0 | 0 | 0.062 | 0.68 | 0.8 | 0.721 | 0.981 |
| TIMP1 | TP53 | 9606.ENSP00000218388 | 9606.ENSP00000269305 | 0 | 0 | 0 | 0 | 0.062 | 0 | 0 | 0.51 | 0.52 |
| TIMP1 | TNF | 9606.ENSP00000218388 | 9606.ENSP00000398698 | 0 | 0 | 0 | 0 | 0 | 0 | 0 | 0.739 | 0.739 |
| TIMP1 | VEGFA | 9606.ENSP00000218388 | 9606.ENSP00000478570 | 0 | 0 | 0 | 0 | 0.085 | 0 | 0.5 | 0.74 | 0.87 |
| TNF | TP53 | 9606.ENSP00000398698 | 9606.ENSP00000269305 | 0 | 0 | 0 | 0 | 0 | 0 | 0 | 0.784 | 0.784 |
| TNF | VEGFA | 9606.ENSP00000398698 | 9606.ENSP00000478570 | 0 | 0 | 0 | 0 | 0 | 0 | 0 | 0.88 | 0.88 |
| TP53 | VEGFA | 9606.ENSP00000269305 | 9606.ENSP00000478570 | 0 | 0 | 0 | 0 | 0 | 0.161 | 0 | 0.81 | 0.834 |
