# Supplementary material for: Investigation of Pharmacological Mechanisms of Yinhua Pinggan Granule on the Treatment of Pneumonia through Network Pharmacology and In Vitro
Source: Biomed Res Int. 2022 Nov 2;2022:1602447. doi: 10.1155/2022/1602447 (PMC9646329; doi:10.1155/2022/1602447)
Supplement: Supplementary Materials — Supplementary Figure legends Supplementary Figure 1: the cluster analysis of GO. the biological process (a), cellular components (b), and molecular function (c) were analyzed by cluster analysis, respectively. Supplementary Table 1 Title: the chemical name of effective components (as noted in alphabets) in Figure 3(a). Supplementary Table 2 Title: the PPI analysis. [file Figure S1.pdf › Figure S1.pdf]

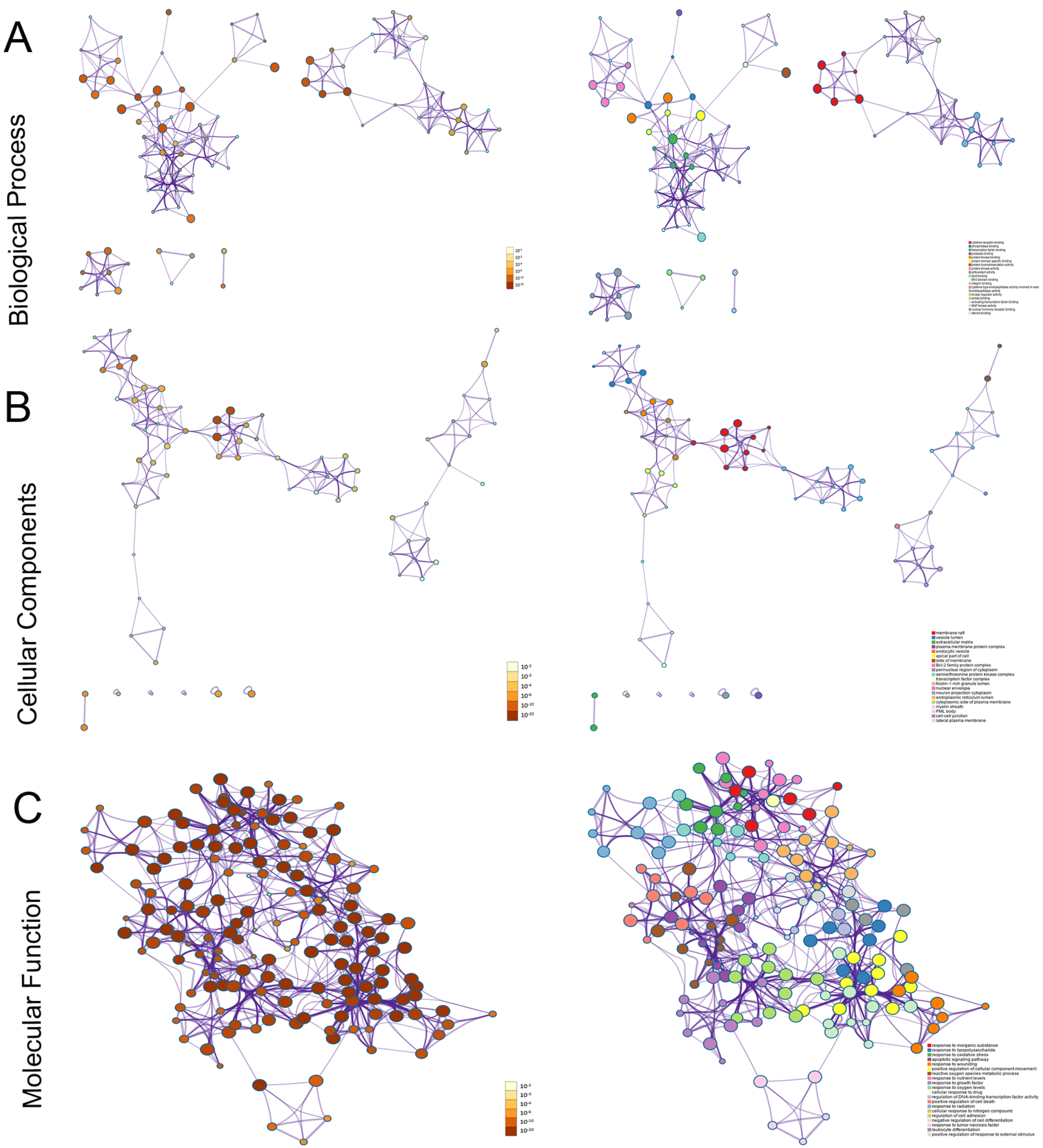
A
Biological Process
10^{-2}
10^{-3}
10^{-4}
10^{-6}
10^{-10}
10^{-20}
cytokine receptor binding
phosphatase binding
transcription factor binding
protease binding
protein kinase binding
protein domain specific binding
protein homodimerization activity
protein kinase activity
antioxidant activity
lipid binding
BH3 domain binding
integrin binding
cysteine-type endopeptidase activity involved in exec
endopeptidase activity
kinase regulator activity
amide binding
activating transcription factor binding
MAP kinase activity
nuclear hormone receptor binding
steroid binding
B
Cellular Components
10^{-2}
10^{-3}
10^{-4}
10^{-6}
10^{-10}
10^{-20}
membrane raft
vesicle lumen
extracellular matrix
plasma membrane protein complex
endocytic vesicle
apical part of cell
side of membrane
Bcl-2 family protein complex
perinuclear region of cytoplasm
serine/threonine protein kinase complex
transcription factor complex
ficolin-1-rich granule lumen
nuclear envelope
neuron projection cytoplasm
endoplasmic reticulum lumen
cytoplasmic side of plasma membrane
myelin sheath
PML body
cell-cell junction
lateral plasma membrane
C
Molecular Function
10^{-2}
10^{-3}
10^{-4}
10^{-6}
10^{-10}
10^{-20}
response to inorganic substance
response to lipopolysaccharide
response to oxidative stress
apoptotic signaling pathway
response to wounding
positive regulation of cellular component movement
reactive oxygen species metabolic process
response to nutrient levels
response to growth factor
response to oxygen levels
cellular response to drug
regulation of DNA-binding transcription factor activity
positive regulation of cell death
response to radiation
cellular response to nitrogen compound
regulation of cell adhesion
negative regulation of cell differentiation
response to tumor necrosis factor
leukocyte differentiation
positive regulation of response to external stimulus
